# An Overview of Bio-Inspired Intelligent Imprinted Polymers for Virus Determination

**DOI:** 10.3390/bios11030089

**Published:** 2021-03-21

**Authors:** Shabi Abbas Zaidi

**Affiliations:** Department of Chemistry and Earth Sciences, College of Arts and Sciences, Qatar University, Doha 2713, Qatar; shabizaidi79@gmail.com or shabizaidi@qu.edu.qa

**Keywords:** molecular imprinted polymers, virus, biosensors, strategies, selectivity

## Abstract

The molecular imprinting polymers (MIPs) have shown their potential in various applications including pharmaceuticals, chemical sensing and biosensing, medical diagnosis, and environmental related issues, owing to their artificial selective biomimetic recognition ability. Despite the challenges posed in the imprinting and recognition of biomacromolecules, the use of MIP for the imprinting of large biomolecular oragnism such as viruses is of huge interest because of the necessity of early diagnosis of virus-induced diseases for clinical and point-of-care (POC) purposes. Thus, many fascinating works have been documented in which such synthetic systems undoubtedly explore a variety of potential implementations, from virus elimination, purification, and diagnosis to virus and bacteria-borne disease therapy. This study is focused comprehensively on the fabrication strategies and their usage in many virus-imprinted works that have appeared in the literature. The drawbacks, challenges, and perspectives are also highlighted.

## 1. Introduction

Viruses are not always the harmful species as they do offer many useful possibilities in biomedical and bionanotechnological applications, anti-microbial agents, synthesis of vaccine, preparation of food materials. Despite their beneficial characteristics, the term viruses can often cause havoc in the general population due to some immensely emphasized deadly diseases such as Corona, Dengue, Avian influenza A H7N9 virus, Severe Acute Respiratory Syndrome (SARS), Hepatitis, Ebola, and Acquired Immunodeficiency Syndrome (AIDS) leading to high mortality and morbidity rates. It can be argued that there are more negative effects than beneficial ones [1,2].

Viruses are responsible for a number of deadly outbreaks in humans and livestock. These diseases may be due to waterborne viruses where the virus is transmitted through the consumption of unsafe and contaminated harmful biological agents. According to the 2017 WHO/UNICEF Joint Monitoring Program (JMP) report, approximately 2.1 billion people do not get clean, readily available water at home, and 6 in 10 or 4.5 billion people do not have access to safe, readily accessible water at home. In addition, the WHO study also points out that at least 2 billion people worldwide use a faeces-contaminated drinking water that is considered, as one of the potential paths of pathogenic human enteric viruses’ infection among masses [3]. According to literature, an infected person can excrete enormous quantities of virus (nearly 105–1011 virus particles/g feces), thus making sewage contamination prevalent. It is therefore very clear to understand that polluted water and inadequate sanitation are associated with the spread of many waterborne outbreaks. Therefore, many efforts have been carried out for increasing access to the de-contaminated water. Some useful methods such as thermal treatment, UV irradiation, and chlorination have significantly used to ensure the availability of safe drinking water. Additionally, some virus such as Dengue virus is spread though mosquitoes which may live on contaminated water [4,5].

Viruses include virions made up of protein coat encapsulated DNA or RNA genome that use various mechanisms to transport genetics to regulate the host cell’s artificial machinery for viral proliferation. Furthermore, since viruses are highly resistant to normal surrounding conditions (i.e., temperatures, pressure, acidic conditions, and disinfectants), they multiply in vast quantities, posing serious threat to healthy organisms. These issues compelled researchers to investigate the fast, effective, and timely detection and diagnosis of viruses for pandemic control and prevention. The literature revealed that there are many outstanding procedures available for the successful diagnosis of virus infections. For example, quartz crystal microbalance (QCM) [6], polymerase chain reaction (PCR) [7], virus-specific IgM antibodies [8], enzyme-linked immunosorbent assay (ELISA) [9], cell culture [10], and electrochemical [11] and photochemical routes [12] have been used routinely; however, the majority of these approaches require expensive antibodies and enzymes for recognition elements apart from complex and cumbersome tasks to perform them, which ultimately make these techniques tedious. Moreover, handling of some methods employing antibodies poses vital issues stemming from the poor stability, short clearance time, and cross-reactivity. Another problem lies in the enormous size of the target and its delicate self-assembled framework in the design of synthetic virus recognition systems [13,14,15]. As a result, the development of efficient, cost-effective, and reusable artificial virus receptors is needed. Denzili group reviewed various bio-sensing strategies for virus detection methods [16]. On the other hand, one interesting review appeared about the utilization of metal-organic frameworks for virus detection, particularly focusing on the coronavirus determination [17].

In the last 2–3 decades, several efforts are being made to prepare synthetic materials in which molecularly imprinted polymers (MIPs) have displayed their peculiar and impressive characteristics, such as their intense affinity, high selectivity, good stability, and reliability in a hostile environment of synthesis and analysis. MIP creates template-shaped cavities in polymer matrices with memory of the template molecules which is similar to “lock and key” method, and one can design a kind of artificial antibody materials that are able to re-interact noncovalently with the imprinted analyte [18,19,20,21,22,23]. In MIP-based papers, an exponential growth has been witnessed, showing many impressive and innovative ways of imprinting a variety of target compounds [24,25,26,27,28,29]. MIPs possess tailor made selectivity, and thus, it is of great interest how the virus imprinted polymers (VIPs) would apply in the identification, classification, and removal of viruses. It may have the potential to provide an effect on a wide array of viral infections, as well as the development of virus-free human pharmaceuticals and consumables. We have seen efforts where attempts are made to prepare and detect protein imprinted polymers successfully albeit by tailoring the synthesis protocols to suit the assays. However, due to their huge size and more intricate surface and spatial configuration, the result is highly cross-linked polymeric matrix and weaker rebinding performance. It is noteworthy that many renowned scientific groups have developed excellent MIP-based sensing for macromolecular targets such as protein and bacteria [30,31,32,33], but there are not as much MIP-based virus sensing strategies. It may be due to difficult handling of viruses, expensive and tedious synthesis or isolation and purification from nature, and less flexible tunable surfaces due to just the presence of DNA/RNA functionality during synthesis. In addition, virus employment in research is tricky in terms of safety and not much labs are equipped with such biosafety clearance levels. Therefore, the development of MIPs for viruses is still a serious hurdle. The improvement in virus imprinting techniques is continually updated amid these complications [34].

There have been a few review papers on virus identification over the last decade. For example, Rijn and Schirhagl [1] discussed the applications of virus particles and artificial virus particles to develop new biomedical applications. Altintas et al. [35] reviewed the biosensors for the detection and removal of waterborne viruses. Afzal et al. [36] reported the review work about QCM based biosensors for early viral diagnostics. Recently, a MIP based viral pathogen related review was also published, but it was just related to human virus infection only [37]. Though the mentioned reviews provide a great deal of insight into virus imprinting, none of these reviews provided the comprehensive account on the imprinting technique utilized in the determination of viral determination. Therefore, we immensely feel the need of a review discussing the progress, methodologies, and challenges in the study of virus imprinting. This work discusses several important articles describing the fabrication of various virus-based sensors and discussing the benefits of prepared sensors so that readers can get the concepts behind these sensors and effective detection strategies. Finally, from the viewpoint of material synthesis and its implementations in viral diagnostic procedures in the near future, the current problems and developmental needs of MIP strategies for virus imprinting are highlighted.

## 2. MIP-Based Virus Sensing Platforms Ordered by Viral Class

### 2.1. Bacteriophage

A bacteriophage, also known as phage, is a virus that infects and reproduces inside a bacterium. The “Fr” is an enteric phage that resides within *Escherichia coli* and is a member of family Leviviridae. The development of a “Fr” bacteriophage imprinted polymer for the prevention of anti-viral infection was demonstrated by Sankarakumar and Tong [38]. For the imprinting, firstly, the whole viral template virus (fr phage) was covalently immobilized onto hydrophobic nanoparticles (made by ethylene glycol dimethacrylate and various functional monomers) monomer followed by redox-initiated miniemulsion polymerization. The resulting imprinted nanoparticles exhibited good anti-viral activity and displayed fast phage titer reduction kinetics within 3 h of contact time. However, the whole process was quite complicated. To overcome the complexity of the procedure, Li et al. [39] developed virus-imprinted MIP on polydopamine-coated silica particles with or without using ammonium persulfate as radical initiator for bacteriophages f2, T4, P1and M13. Due to the imprinted polymers, noteworthy dose-dependent and time-dependent suppression of virus infection in host cells was noticed within 12 h. Furthermore, the proposed MIPs were biocompatible and non-toxic with excellent stability and reusability. Dopamine was chosen due to its biocompatibility, hydrophilic behavior, and self-polymerization capability. It was observed that addition of ammonium persulfate increased the imprinting effects and induced dopamine polymerization nearly around isoelectric point of f2 phage (pH-6), unlike dopamine self-polymerization at basic pH with it. The improved imprinting may be due to dopamine polymerization at pH = 6, which helps to preserve the structural stability of the f2 phage.

Altintas et al. [40] proposed a highly efficient MIP-based surface plasmon resonance (SPR) biosensor using a new solid-phase synthesis method to detect bacteriophage MS2. To prepare the sensor, firstly, template-derivatized glass beads were synthesized followed by synthesis of MIP nanoparticles. The characterization showed that the size of nanoMIPs with spherical structural morphology varying from 200 to 230 nm was obtained. As plaque-forming units (pfu) with excellent regenerative ability, a significantly higher affinity between the synthetic ligand and the target bacteriophage MS2 was found to be about ∼3 × 10^−9^ M with a LOD value of 5 × 10^6^ pfu·mL^−1^. In addition, cross-reactivity evaluation with other viruses or analytes such as vancomycin and QB phage using the SPR sensor demonstrated the promising behavior of nanoMIP for selective virus determination.

### 2.2. Adenovirus

Adenoviruses are medium-size virus causing cold, bronchitis, diarrhea, and urinary bladder infection, etc. They are composed of double-stranded DNA genome. Altintas and co-workers [41] developed adenovirus imprinted SPR sensor. It performed well in the linear concentration range of 0.02 to 20 pM, with a LOD of 0.02 pM. As an alternative to MIP receptors, direct and sandwich assays were developed for adenovirus quantification using natural antibodies with the LOD values of direct and sandwich assays of 0.3 pM and 0.008 pM, respectively. The group of Mizaikoff [42] investigated the use of bovine serum albumin (BSA) for the reduction of unspecific binding (passivation agent) in adenovirus imprinted silica micro-sized particles via sol–gel method. Binding assays conducted in PBS with the inclusion of BSA were capable of binding up to 97% of the incubated virus as compared to just 5% binding on non-imprinted particles. It also showed good competitive selectivity toward adenovirus as compared to minute virus of mice (MVM). In addition, this developed method is universal and can be applied with appropriate choice of sol–gel reaction conditions to a broad range of other biological systems. Gast et al. [43] developed a strategy where the viral hexon protein, (most abundant and accessible surface protein component of the human Adenovirus type 5 (hAdV5)) was used as template. It created cavity in the presence of suitable polymer conditions and was able to recognize the entire hAdV5. The proposed biosensor exhibited higher selectivity for hAdV5 than MVM.

### 2.3. Dengue Virus

The Dengue virus, one of the most feared mosquito-borne viruses, specifically targets humans and primates and can cause a particular form of hemorrhagic fever called dengue fever. It has shown its deleterious effects in developing countries since last decade. The Dengue fever demands urgent and accurate diagnosis due to its rapid and fatal infections just after 4 days of fever onset. Furthermore, 1 of the 4 antigenically different dengue virus types is the main cause of infection and the infection from virus does not provide cross-protective immunity against the others. Therefore, practitioners need to be particularly vigilant in order to diagnose the difference between primary and secondary dengue virus infections [44,45,46].

The first MIP based imprinting for dengue virus was performed by Tai group [47], where a epitope of dengue protein NS1 called pentadecapeptide (15-mer peptide, (1613 Mw) was imprinted in the presence of a monomer solution, containing acrylic acid/acrylamide/*N*-benzylacrylamide via UV irradiation onto a QCM chip. The average thickness of polymeric thin film was estimated to be 70 nm. The comparison between epitope imprinted polymer and protein surface imprinting polymer showed that both the template and the proteins (including mother protein NS1 of 24,000 Mw) that had the same epitope part of the structure were effectively identified by the polymers imprinted with a peptide. This approach achieved a corresponding frequency change in QCM chips immobilized with Dengue virus monoclonal antibodies. To realize the application of above-mentioned method, Tai group [48] tested the affinity of their epitope-imprinted films in dengue affected patients’ serum samples. Based on the comparison of this study with traditional ELISA technique, it is worth mentioning that the correlation coefficient of the QCM response and the ELISA result was 0.73 with acceptable repeatability and reproducibility between 4–28% and 10–32%, respectively. To cater the demand of commercial MIP product, Lieberzeit et al. [49] demonstrated the synthesis of Dengue virus imprinted polymers by incorporating the MAA and NVP and EGDMA, as functional monomer and cross-linker, respectively and tested as QCM sensor. The results indicated promising sensor response.

### 2.4. Influenza A Virus

The avian virus poses a serious threat to mammals, including humans, to a large degree, as common avian virus variants can lead to avian H5N1 strains that are more likely to infect mammals widely [50,51]. Among 5 common virus strains (H5N1, H5N3, H1N1, H1N3, and H6N1), H5N1 is considered the main culprit for inducing infections in humans [52]. Hence, MIP technique is employed for the determination of specific virus strain in some research works.

**Figure 1:**
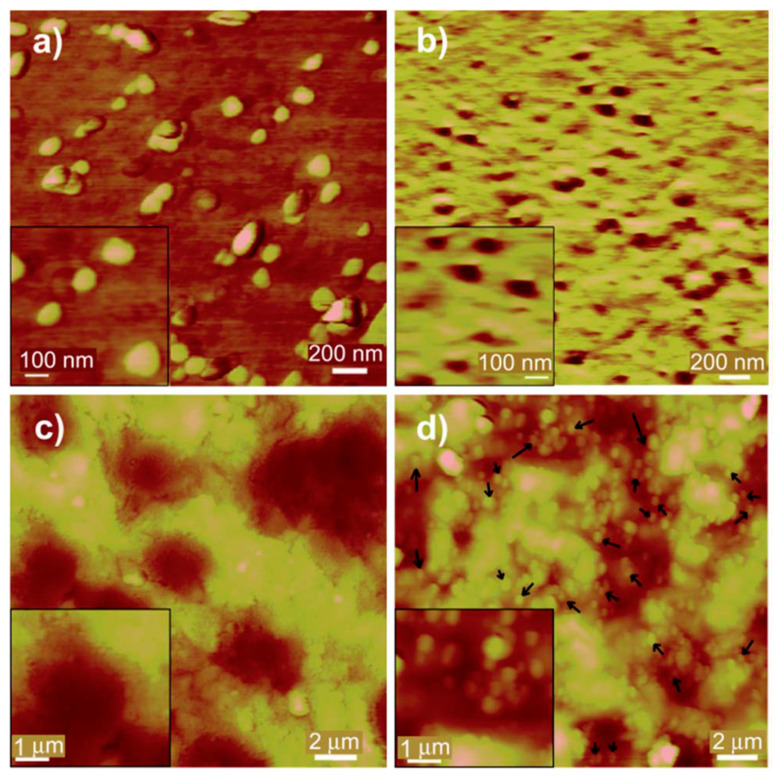
AFM images of (**a**) the H1N3 influenza A virus used as a template, (**b**) the corresponding H1N3 based MIPs at 200 nm resolution. The MIP cavities in the latter have been found to be between 80 and 120 nm in diameter, which is the expected size of influenza A. Also illustrated is the structure of the MIP with (**c**) and without template bound (**d**) displayed at 2 mm resolution. The white spots present in the template bound structure (**d**) are indicative of bound virus (some bound virus is marked by dark arrows) (Reproduced with permission from Reference [53]).

Wangchareansak and co-workers [53] were the first to imprint the 5 different strains of Influenza A virus in the presence of various monomer mixture including acrylamide (AM), methacrylic acid (MAA), methylmethacrylate (MMA), N-vinylpyrrolidone (VP), N,N-(1,2-dihydroxyethylene) bisacrylamide as cross-linker, and other constituents required for polymer synthesis via stamp and spin coating on gold electrode and analyzed by QCM technique. QCM is capable of exhibiting distinction between subtypes of influenza A that have similar structures but differ in the number of different surface amino acids. The use of various monomers provides both polar and hydrophobic groups capable to attach well on protein surfaces in Influenza A virus. It was noteworthy that co-polymers consisting of AAM, MAA, and MMA could not distinguish the 5 different virus sub-types; however, selectivity was significantly improved by the addition of VP. The AFM results in Figure 1 show the successful imprinting, removal, and rebinding processes with corresponding size distribution of the influenza.

A. Selectivity studies indicate that MIPs were selective towards the corresponding virus template and LOD as low as 105 particles·mL^−1^ was achieved.

**Figure 2:**
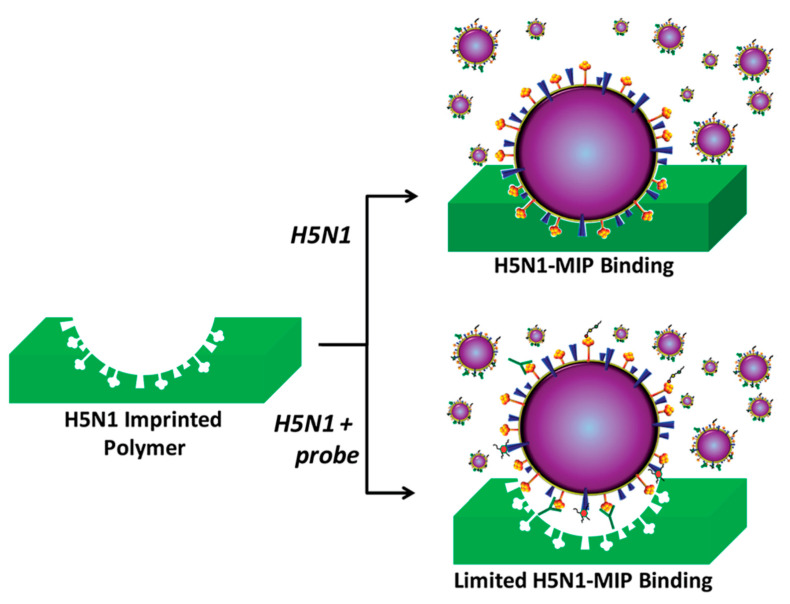
Schematic of H5N1 virus binding to a MIP (**top**) and inhibition of the process due to a conformational change in the H5N1-probe complex (Reproduced with permission from Reference [54]).

The same group [54] used the same Influenza imprinted H5N1 strain polymer with various probes like anti-H5 and anti-H1 anti-influenza A hemagglutinin (HA) antibodies, derivatives of sialic acid and N-acetylglucosamine (GlcNAc), parts of the influenza A receptor targeting the HA receptor binding pocket protein, and the anti-neuraminidase drug oseltamivir. According to the hypothesis called allosteric mechanism used in this work, when a probe molecule binds to the virus, the virus uptake on the H5N1 MIP should reduce, thus blocking it from attaching to the MIP, as shown in Figure 2. 

The binding of H5N1 to the polymer was reported to be strongly inhibited by oseltamivir compared to anti-H5, which in turn is more effective than sialic acid. These experimental findings are promising because they can discern conformational effects from small inhibitors and substrates to macromolecules. Despite the good selectivity, both of these methods were tedious in terms of preparation and assay of the imprinted polymer. Karthik et al. [55] suggested a simple way for imprinting the Influenza A (HK68) using polydimethylsiloxane (PDMS). The resulting polymers consisted of cavities with an average size of 120 ± 4 nm. The imprinted cavities exhibited rapid (within 1 min) affinity toward the target virus in trace aqueous suspension (5 μL) and provided superior LOD of 8 fM. The same technique has also been applied for Newcastle Disease Virus (NDV) imprinting. Influenza A (HK68) imprinted polymer binds specifically to HK68 at a capture ratio of 1:8.0 compared to NDV imprinted polymer, which displayed a capture ratio of 1:7.6 in fluorescently labeled NDV and HK68 mixtures.

### 2.5. Poliovirus

Poliovirus is one of the most harmful viruses causing poliomyelitis, a crippling and potentially deadly infectious disease, which mainly affects young children. The disease causes partial paralysis that is often permanent and cannot be cured [56]. Hence, immunization is necessary to prevent the polio infection. Wang et al. [57] demonstrated the fabrication of poliovirus imprinted potentiometric sensor. The co-templates carcinoembryonic antigen (CEA) and poliovirus were self-assembled imprinted with the help of hydroxyl-functionalized alkanethiol onto a gold-coated silicon. During analysis, the removal or re-adsorption caused the fluctuation in potential owing to electrical manipulation ability of virus. The maximum concentration of the poliovirus sensor test estimated to be ∼3100 × 10^8^ virus particles·mL^−1^ for poliovirus that was comparable to template solution of 3880 × 10^8^ virus particles·mL^−1^ (0.644 nM).

### 2.6. Hepatitis A Virus (HAV)

**Figure 3:**
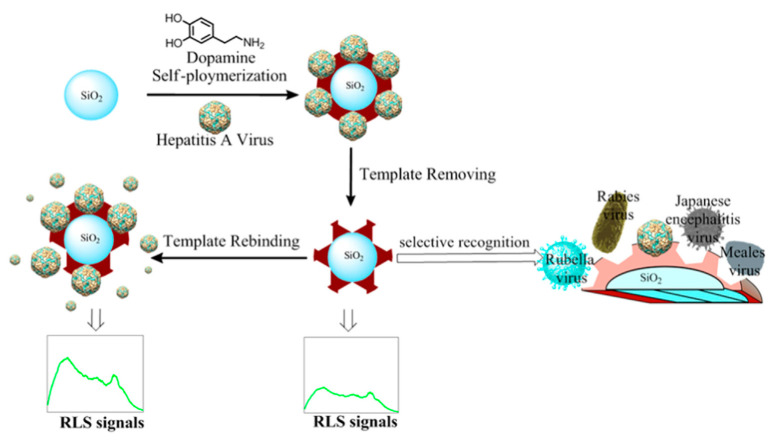
Principle of preparation of the virus-MIPs and detection of virus (Reproduced with permission from Reference [59]).

HAV is responsible for serious liver disease by consumption of contaminated foods and water or by direct contact with an infected individual. Acute HAV infection is hard to differentiate from other types of Hepatitis infections and influenza infection, thus accurate and specific diagnosis is a needed [58]. Cai group [59] developed a resonance light scattering (RLS) sensor using polydopamine (PDA)-coated HAV-imprinted polymer on the surface of SiO_2_ nanoparticles via one-step synthesis method as shown in Figure 3. Figure 3 shows that cavities corresponding to HAV were formed, indicating that the fabricated polymer was highly selective against HAV only in comparison to other viruses like Rubella, Rabies, JEV, and measles virus.

Improved RLS intensity corresponding to HAV concentration from 0.04 to 6.0 nmol∙L^−1^ and LOD of 8.6 pmol∙L^−1^ were obtained. The sensor was very selective toward HAV compared to other viruses such as rabies vaccine, Japanese encephalitis (JEV), and the mixture of measles vaccine and rubella vaccine. In addition, it could identify HAV from a 20,000-fold diluted human serum sample. Cai and coworkers [60] synthesized CdTe/CdS quantum dot (QD) modified silica nanoparticles using sol-gel process for selective imprinting of HAV. The proposed sensor exhibited LOD of 88 pM with linear working range between 0.2 to 1.4 nM via fluorescence quenching. The sensor was selective toward HAV as compared to another structurally similar virus species like JEV, HBV, and Rabies virus. It achieved satisfactory recoveries of HAV from 96.7 to 101.6% from spiked human serum samples.

Recently, Cai and co-workers [61] fabricated an interesting pH responsive HAV imprinted polymer nanoprobes using Material Institut Lavoisier-101 (MIL-101) particles in a metal-organic framework. In this work, dimethylaminoethyl methacrylate (DMA) was responsible to adjust the pH for capturing and releasing the HAV followed by determination by RLS technique. It exhibited a linear concentration of 0.02–2.0 nM and an excellent LOD of 0.1 pmol·L^−1^ with in 20 min. A reasonable recovery of 88% to 108% of HAV from HAV spiked human serum samples was obtained. The determination of HAV and HBV (Hepatitis B virus) is challenging owing to their similar structures. Hence, the Cai group [62] proposed another good approach where visual simultaneous determination of HAV and HBV was realized via red and green colored quantum dots (R-CdTe Quantum Dots & G-CdTe Quantum Dots) using different concentrations of HAV and HBV. As per the performance of the sensors, it has been reported that nonspecific binding was significantly decreased and selectivity was improved due to the utilization of hydrophillic monomers and metal-chelation. Excellent LODs of 3.4 and 5.3 pmol/L for HAV and HBV, respectively, were obtained within 20 min.

### 2.7. Japanese Encephalitis Virus (JEV)

JEV is a mosquito-borne zoonotic pathogen of severe concern because it causes Japanese Encephalitis (JE), which is a neurotrophic killer disease, which, in turn, is responsible for viral acute encephalitis syndrome (AES) globally. Cai group worked in this area and reported 2 different protocols to determine JEV via imprinting method.

In their first report, a surface imprinted fluorescent sensor using JEV with magnetic silicone micro-spheres acting as carrier materials and tetraethyl orthosilicate (TEOS) and 3-aminopropyl triethoxysilane (APTES) functioning as building blocks was proposed. The sensor provided an imprinting factor of 2.98. Moreover, sensitive fluorescent detection in water with a good linearity within 2.5–45 nM, and a detection limit of 0.32 nM with excellent selectivity of JEV in 1000-fold dilution of human serum sample solution was achieved [63]. JEV imprinted polymers were functionalized over silica microspheres (SiMPs) using APTES in another approach, which was modified by fluorescent dye, pyrene-1-carboxaldehydehyde (PC). The PC was employed based on the fact that the fluorescence intensity of PC can be enhanced by the virus, where the virus and PC were used as energy donor and energy acceptor, respectively in fluorescence resonance energy transfer (FRET) mechanism. The sensor displayed linear response in the 24–960 pM range, with a 9.6 pM LOD under % RSD for the virus solution. This sensor was able to recognize JEV in the 2000-fold human serum sample dilution [64].

In order to synthesize JEV imprinted fluorescence sensor on silicon-modified metal organic frameworks using zinc acrylate as a functional monomer, the Chen and Cai group [65] explored free radical polymerization. A passivating agent, polyethylene glycol (PEG), was used as a blocking agent to improve the selectivity of JEV. This sensor offered a LOD of 13 pM and performed linearly between 50 pM to 1400 pM. Moreover, due to good imprinting factor of 4.3, a recovery rate of JEV between 92.50% and 114.35% was obtained in spiked human serum samples. This group fabricated JEV imprinted magnetic polymer particle sensor and analyzed by using RLS. The magnetic polymers particles were easily separated after analysis. The proposed sensor analyzed time was 20 min and it exhibited LOD of 1.3 pM toward JEV with high selectivity in the presence of HAV, Simian virus 40 (SV40) and Rabies virus [66]. Gong and Cai group [67] developed another JEV imprinted magnetic fluorescence sensor using Dansyl chloride (DNS-Cl) that exhibited excellent fluorescence properties. For the sensor fabrication, DNS-Cl was covalently immobilized on SiMPs followed by JEV imprinting in the presence of APTES and TEOS. A linear fluorescence intensity from 2.4 to 24 pmol·mL^−1^ and 0.11 pM of LOD was achieved for JEV. The sensor showed larger quenching toward JEV as compared to other virus species such as HAV, SV40, and Rabies virus and offered recoveries between ~98–100.56% in spiked human serum samples.

### 2.8. Apple Stem Pitting Virus (ASPV)

**Figure 4:**
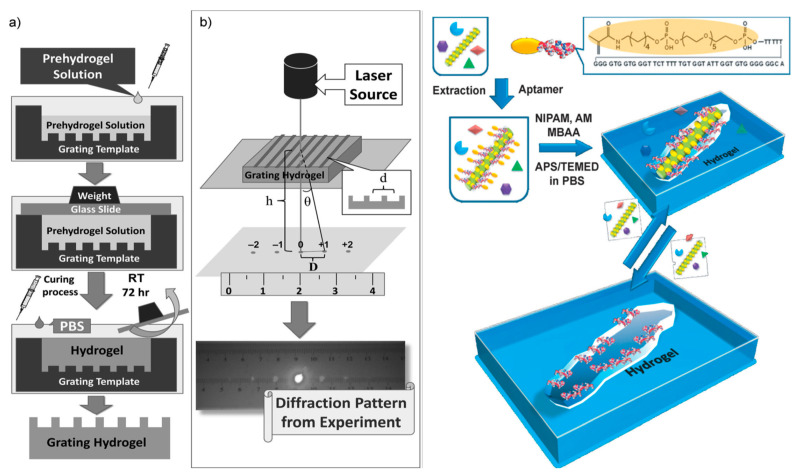
(**a**) Preparation of “double imprinted” diffraction grating bioimprinted hydrogels for MIP-GLaDiS materials. (**b**) Schematic of the laser diffraction apparatus used for the measurement of the laser diffraction pattern projected onto a desk ruler (left). Outline of the bioimprinting process used to create virus responsive super-aptamer hydrogels. NIPAM = N-isopropylacrylamide, AM = acrylamide, MBAA = N,N’-methylene bisacrylamide, APS = ammonium persulfate, TEMED = N,N,N’,N’-tetramethylethylendiamine, PBS = phosphate-buffered saline (right) (Reproduced with permission from Reference [68]).

Apple stem pitting virus (ASPV), a widespread plant pathogenic virus. Via a dual imprinting method, Bai and Spivak [68] developed a visual sensor in which a virus imprinted hydrogel is micromolded into a diffraction grating sensor utilizing imprint-lithography technology to achieve a “Molecularly Imprinted Laser Diffraction Sensor Polymer Gel” (MIP-GLaDiS) as shown in Figure 4.

The sensor was capable of detecting the ASPV by simple laser transmission equipment up to 10 ng·mL^−1^, which was in close agreement to the results obtained from ELISA or fluorescence-tag systems.

### 2.9. Picornaviruses

Some of the significant human and animal pathogens belong to the picornavirus virus family. For instance, human rhinovirus (HRV) or the foot-and-mouth-disease virus (FMDV) are two typical examples of Picornaviruses and they have common icosahedral shape with an outer protein capsid and an RNA strand in their centers. There are nearly 100 major stereotypes of HRV. Therefore, synthetic recognition materials are required not only to determine HRV, but also to distinguish between different serotypes.

**Figure 5:**
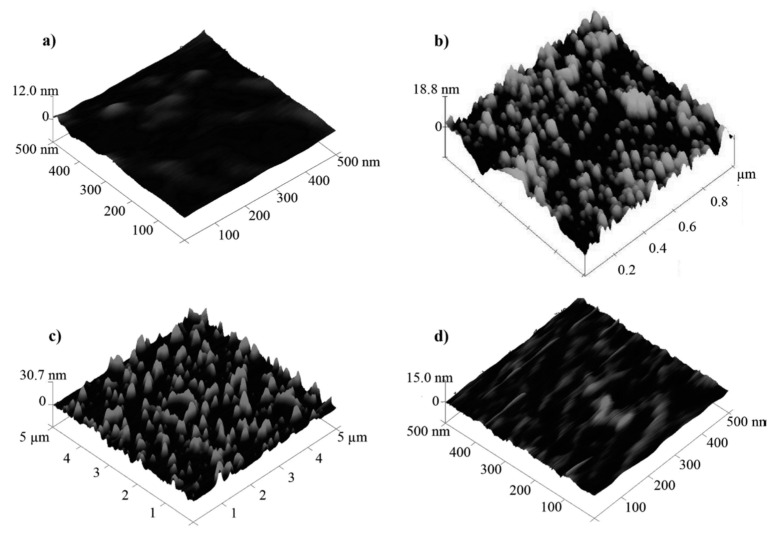
Contact mode AFM images of polyurethane layers: (**a**) nonimprinted polymer, (**b**) human rhinovirus (HRV) self-assembled on a surface, (**c**) molecular imprinting polymer (MIP) with partially removed template, and (**d**) MIP after washing (Reproduced with permission from Reference [69]).

Jenik et al. [69] exploited stamp imprinting to prepare HRV patterned polyurethane film as conformed by AFM technique shown in Figure 5.

The QCM measurements demonstrated that sensor yielded excellent signal (−300 Hz) in a virus suspension of ∼100 μg·mL^−1^. Furthermore, the imprinted cavities were capable to differentiate the various stereotypes and types of virus with high selectivity. Hussein et al. [70] prepared FMDV serotype O imprinted sensor using electro-polymerization of the oxidized O-aminophenol film on a gold screen-printed electrode. The developed biosensor exhibited LOD of 2.0 ng·mL^−1^ that was 50 times lower than the ELISA and PCR in the analysis of saliva real samples. The biosensor offered good selectivity toward FMDV serotype O over all other genus serotypes A, SAT2, Lumpyskin disease virus (LSDV), and inactivated serotype O.

### 2.10. Turnip Yellow Mosaic Virus (TYMV)

**Figure 6:**
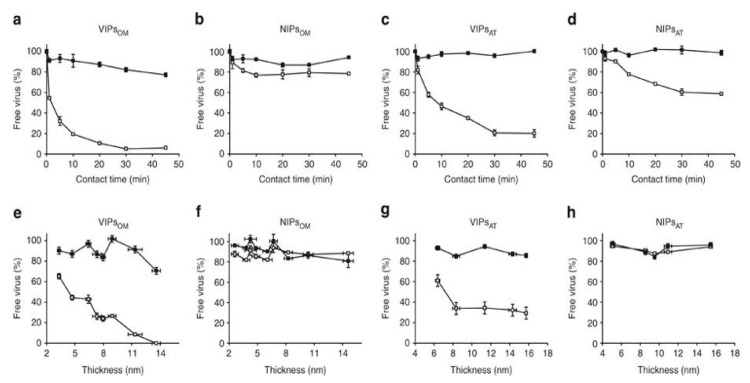
Binding of virions of the templated turnip yellow mosaic virus (TYMV) and non-templated tomato bushy stunt virus (TBSV) to virus imprinted polymers (VIPs) and non-imprinted particles (NIPs). Symbols are for TYMV (open squares) and for TBSV (solid squares). Binding time, selectivity, composition and thickness of the recognition layer were compared. (**a**–**d**) Four types of particles were assayed: (**a**) VIPs_OM_, (**b**) NIPs_OM_, (**c**) VIPs_AT_, and (**d**) NIPs_AT_; OM and AT particles with 8-mm-thick recognition layers. (**e**–**h**) Nanoparticles with recognition layers of increasing thicknesses (mean ± s.e.m.) were assayed: (**e**) VIPs_OM_, (**f**) NIPs_OM_, (**g**) VIPs_AT_, and (**h**) NIPs_AT_. All values are presented normalized in percentage of initial virus concentration (mean ± s.e.m.)(The term VIPs_OM_ stands for particles imprinted with TYMV virions and having a recognition layer composed of an organosilanes mixture (OM), and the term non-imprinted particles (NIPs)_OM_ for NIPs produced in the absence of template using the same OM. As controls, we selected two additional formulations, one with tetraethyl orthosilicate (TEOS) alone and one with a mixture of APTES and TEOS (AT). The corresponding VIPs are abbreviated VIPs_AT_ for TYMV imprinted particles having a recognition layer made of AT and NIPs_AT_ for those produced under the same conditions in the absence of template) ((Reproduced with permission from Reference [72]).

Tomato bushy stunt virus (TBSV) and turnip yellow mosaic virus (TYMV) are non-enveloped, icosahedral, single-stranded RNA plant viruses. Cumbo and co-workers [71,72] targeted these two plant viruses to demonstrate the fabrication of highly selective imprinting cavities via surface imprinting. The imprinting procedure was composed of 3 steps where, firstly, the virus was bound to silica nanoparticles (SNPs), and then, the virus-modified nanoparticles were incubated in a mixture of organosilanes to grow via polycondensation, and in the last step, the virus was removed to create the imprint recognition layer. Figure 6 clearly reveals the binding of virions of the templated TYMV and non-templated TBSV to VIPs and NIPs in batch-rebinding assay. The results obtained were in close agreement with ELISA test.

### 2.11. Tobacco Mosaic Virus (TMV) and Tobacco Necrosis Virus (TNV)

A well-studied and characterized plant virus, tobacco mosaic virus (TMV) is discussed in this section. Many researchers used TMV as a model virus for their studies due to its robust nature, which can resist any conformation and activity change even after being subjected to harsh environmental conditions [73].

Hayden et al. [74] explored stamping method for the synthesis of TMV imprinted polymer over amylose layer using gold QCM electrode. The imprinted films showed high binding affinity toward TMV as compared to negligible response with NIP. The Dickert group [75,76] used two polymers namely polyurethanes and polyacrylates in another work. To avoid the participation of TMV in polymerization in polyurethanes, a thin layer of glucose solution was formed over TMV viruses on the self-organized template stamp. On the other hand, by spin coating, acrylate materials could be prepared in aqueous buffered solutions, enabling the monomers to be combined with the template, thus producing a self-assembly method to the sensing substrate. The sensor response of imprinted and non-imprinted polyurethanes to a 1 mg·mL^−1^ TMV solution clearly showed the impact of imprinting. Moreover, acrylate polymer layer performed better than the polyurethane polymer layer. The imprinted polymers exhibited good selectivity toward TMV in the presence of human rhinovirus serotype-2 (HRV-2) as observed in QCM studies.

Bolisay et al. [77] followed simple protocol to synthesize TMV imprinted hydrogel via non-covalent synthesis using poly(allylamine hydrochloride) (PAA-HCl) as the polymer matrix. TMV-imprinted hydrogels displayed improved TMV binding (8.8 mg TMVg^−1^ polymer) relative to non-imprinted hydrogels (4.2 mg TMV g^−1^ polymer) in batch experiments. In another study, Bolisay and co-workers [78] improved their TMV imprinting work that prevented aggregation of templates and increased removal of templates. The results revealed that more than 25% *w*/*v* poly(allylamine hydrochloride) (PAA)/TMV) concentration avoided the polymer-virus aggregation as compared to less than 0.0001% *w*/*v* concentration. Secondly, 1 M NaOH performed better among several washing methods, such as H_2_O, 1 M NaCl, 1 M NaOH, and 6 M Urea. Optimized TMV MIPs synthesized by these findings exhibited a strong affinity to TMV (printing factor 2.3) and a poor binding to tobacco necrosis virus (TNV), the non-target virus. In the presence of a template virus in an optimized protocol, Bolisay and Kofinas [79] used more versatile non-covalent imprinting hydrogels using polyallylamine (PAA) as a monomer with ethylene glycol diglycidyl ether (EGDE) as cross-linker. A PDMS microfluidic chip consisting of reaction and reference channels containing four contactless dielectric microsensors made with either native or MIP was created by Birnbaumer and co-workers [80] for continuous detection of TMV viral contamination. A virus stamped imprinted film of 200 nm thickness was synthesized co-polymer comprised of MAA and VP via spin coating. The use of chip is useful as manipulated electrics of viruses, detection, and identification were carried out simultaneously using dielectric spectroscopy, dielectrophoresis, and impedance, respectively. During impedance experiments, both native and imprinted-polymers showed significant resistance increase in the presence of TMV when 100-fold dilution or 40 mg·mL^−1^ TMV solution with 35 mm·s^−1^ fluid flow was used. This method offered excellent sensitivity until 4 mg·mL^−1^ (1000-fold decrease) of TMV solution and the imprinted MIP cavities could successfully differentiate HRV2 samples despite similarities in TMV and HRV stereotypes. 

To overcome the hurdles of polymer-virus aggregation and virus removal from polymer matrix, Ikawa and coworkers [81] immobilized TMV over azobenzene functionalized acrylate polymer followed by imprinting in the presence of suitable monomer solutions. The efficient immobilization with successful determination of virus with MIP surfaces were assessed by atomic force microscopy and immunological enzyme luminescence, respectively. The tobacco necrosis virus (TNV) is another plant pathogen virus that is often spread from contaminated water. TNV is spherical and has a size of nearly 20 nm. Wankar and colleagues [82] demonstrated the imprinting of TNV by using electropolymerization of polythiophene film with an approximate thickness onto conducting Au surfaces. Later, fluorescence intensity of 410 nm exhibited response for TNV between 0.1–10 ng·L^−1^ (0.15–15 pg) and exhibited a LOD of 2.29 ng·L^−1^ (3.4 pg). The response time was just 2 min with high selectivity as negligible fluorescence response for TMV obtained for 10 ng·L^−1^ sample solution. Moreover, the sensor showed satisfactory recovery of TNV in spiked water samples with standard deviation less than 3.5%.

### 2.12. Norovirus

Human Noroviruses are known for causing severe gastroenteritis infections [83]. The corresponding virus-like particles (VLPs) were used systematically to examine the structure and stability of the virus, infected cell interactions, and as diagnostics tools. Thus, Sykora et al. [84] used a VLP of Norovirus (NorVLP) obtained from predominant genotype II strain 4 (GII.4) in the imprinting process by immobilization it on amino-SNPs using glutaraldehyde as homo di-functional cross linker. This was followed by a self-assembly of silane and polycondensation reactions forming an organosilic detection layer using APTES and TEOS as building blocks. Binding studies found that 80% of NorVLP was attached to imprinted polymers (in only 30 min, compared to just 30% for NIPs).

### 2.13. Swine Flu Virus

Classical swine fever (CSF) is one of the extremely infectious viral diseases caused by classical swine fever virus (CSFV), a member of the *Pestivirus* genus. The World Organization for Animal Health (OIE) has categorized this as a threat to the international export of pork around the globe [85]. Klangprapan et al. [86] developed a simple CSFV imprinted copolymer comprising MAA, VP, acrylamide (AAM), and methyl methacrylate (MMA) as monomers and analyzed via QCM. The SEM characterization showed that the diameter of the obtained imprinted cavities was ~59 nm similar to the dimension of CSFV. The proposed sensor exhibited LOD of 1.7 μg.mL^−1^ and the selectivity factors of 2 over porcine respiratory and reproductive virus (PRRSV) and 62 over pseudorabies virus (PRV) were achieved.

### 2.14. Zika Virus

Zika virus (ZIKV) is a member of the *Flaviviridae* family. Generally, it is not known to be much dangerous, but recent reports of a heightened incidence of microcephaly in infants born to women infected with ZIKV during pregnancy have led to concern in the identification and analysis of ZIKV. Tancharoen et al. [87] developed an interesting and simple approach for the ZIKA determination. In this work, prepolymer gel was prepared by using a mixture of four monomers including AAM, MAA, MMA, and VP followed by mixing with Graphene oxide (GO) to obtain a composite. The composite was drop coated over gold electrode by spinning, and later, ZIKA was dispersed over it followed by exposure to UV light for the polymerization. In phosphate buffer, the biosensor recognized ZIKV upto 2 × 10^−4^ pfu·mL^−1^ (1 RNA copy·mL^−1^). In addition, the LOD in the presence of dengue virus was eastimated to be 2 × 10^−2^ pfu·mL^−1^.

## 3. Challenges and Future Perspectives

In the above section, we have comprehensively discussed various fabrication approaches for the accurate, selective, and sensitive determination of viruses. Despite many exemplary ways, the road of virus detection seems full of hurdles and challenges. For example, techniques which do not involve extensive equipment or specialized staff are desperately needed, particularly if the findings can be interpreted by the unaided eye, which could significantly lower the cost of manufacturing. Unfortunately, there are only a few works performed where visual detection of the virus was explored (68). Miniaturization and portability are another crucial need of the time when patients may be given point-of-care diagnosis. The incomplete removal of the polymer matrix and sluggish mass transfer kinetics is one of the most significant problems in large imprinting biomolecules. Using surface imprinting, these problems were alleviated, but their solutions introduced laboriousness and arduousness into the fabrication processes [30,88]. Some researchers tried easy ways to imprint viruses via electropolymerization of conducting polymers. Ultimately, we still require some facile and robust ways for virus diagnosis via imprinting techniques. As discussed in the preceding section, MIP hydrogels were also prepared for the detection of viruses successfully; nevertheless, their behavior in swollen condition and maintaining the strict shape of imprinted cavities are still the cause of worry. The chemical reactivity of monomers with viruses is one of the challenges during virus printing, and a protective layer was therefore added to the surface of the virus prior to printing.

During the current COVID-19 crisis, one of the biggest challenges is the accurate and reliable COVID-19 diagnostics. While there have been reports, even commercial efforts, that highlight the use of immunosensors to create fast test alternatives for the routinely used PCR analysis [89,90,91], no such real time MIP-based COVID-19 diagnostic is available. As discussed before in the introduction section, lab biosafety concern, less functional groups over the viruses’ surface, various mutants of COVID-19, and isolation or purification of COVID-19 virus made it more difficult to develop highly selective COVID-19 imprinted sensors.

Despite many challenges ahead, the MIP technique is attractive and increasingly utilized in virus imprinting as it is evident from many works that yield highly selective customized cavities. They are cost effective and could stand a harsh environment with outstanding stability and reusable artificial receptors. We could safely assume that MIP is going to play pivotal role in virus diagnosis.

## 4. Conclusions

We have summarized several recent achievements and efforts in advancing bio-inspired imprinted polymers for virus identification in this work. The latest advancements demonstrate that the techniques for determining trace amounts, early diagnosis, and removal of viruses from imprinted polymers have evolved enormously. It obviously enhanced the understanding and knowledge of the production of various types of virus sensing and removal methods with sensors including quartz crystal microbalance, electrochemical, fluorescence, batch rebinding experiments, surface plasmon resonance methods, and so on. It should be noted that MIP-based methods, despite many associated challenges, have several important advantages that are sufficient to promote the growth of virus-determining strategies. Finally, there is a tremendous demand to develop the commercially diagnostic and point-of-care kits for virus detection and removal, which will ultimately lead to the realization and actual advancement in medicine and healthcare benefits. Only then, we will be able to enjoy their benefits in medical and other related areas.

## Data Availability

This reviews follows the “MDPI Research Data Policies”.

## References

[1] Van Rijn P., Schirhagl R. (2016). Viruses, artificial viruses and virus-based structures for biomedical applications. Adv. Healthc. Mater..

[2] Singhal C., Pundir C.S., Narang J. (2017). A genosensor for detection of consensus DNA sequence of Dengue virus using ZnO/Pt-Pd nanocomposites. Biosense. Bioelectron..

[3] Nidzworski D., Siuzdak K., Niedziałkowski P., Bogdanowicz R., Sobaszek M., Ryl J., Weiher P., Sawczak M., Wnuk E., Iii W.A.G. (2017). A rapid-response ultrasensitive biosensor for influenza virus detection using antibody modified boron-doped diamond. Sci. Rep..

[4] Kumar P.K.R. (2016). Monitoring Intact Viruses Using Aptamers. Biosensors.

[5] Kizek R., Krejcova L., Michálek P., Rodrigo M.M., Heger Z., Krizkova S., Vaculovicova M., Hynek D., Adam V. (2015). Nanoscale virus biosensors: State of the art. Nanobiosens. Dis. Diagn..

[6] Wang R., Wang L., Callaway Z.T., Lu H., Huang T.J., Li Y. (2017). A nanowell-based QCM aptasensor for rapid and sensitive de-tection of avian influenza virus. Sens. Actuators B.

[7] Clem A.L., Sims J., Telang S., Eaton J.W., Chesney J. (2007). Virus detection and identification using random multiplex (RT)-PCR with 3′-locked random primers. Virol. J..

[8] Nagar P.K., Savargaonkar D., Anvikar A.R. (2020). Detection of Dengue Virus-Specific IgM and IgG Antibodies through Peptide Sequences of Envelope and NS1 Proteins for Serological Identification. J. Immunol. Res..

[9] Chen L., Ruan F., Sun Y., Chen H., Liu M., Zhou J., Qin K. (2019). Establishment of sandwich ELISA for detecting the H7 sub-type influenza A virus. J. Med. Virol..

[10] Leland D.S., Ginocchio C.C. (2007). Role of Cell Culture for Virus Detection in the Age of Technology. Clin. Microbiol. Rev..

[11] Chowdhury A.D., Takemura K., Li T.-C., Suzuki T., Park E.Y. (2019). Electrical pulse-induced electrochemical biosensor for hepatitis E virus detection. Nat. Commun..

[12] Guliy O., Kanevskiy M., Fomin A., Staroverov S., Bunin V. (2020). Progress in the use of an electro-optical sensor for virus detection. Opt. Commun..

[13] Caygill R.L., Blair G.E., Millner P.A. (2010). A review on viral biosensors to detect human pathogens. Anal. Chim. Acta.

[14] Abdelghani A., Serra P.A. (2011). Electrochemical Biosensors for Virus Detection. Biosensors for Health, Environment and Biosecuri-Ty.

[15] Daaboul G.G., Lopez C.A., Yurt A., Goldberg B.B., Connor J.H., Unlu M.S. (2012). Label-free optical biosensors for virus detec-tion and characterization. IEEE J. Sel. Top. Quantum Electron..

[16] Saylan Y., Erdem Ö., Ünal S., Denizli A. (2019). An Alternative Medical Diagnosis Method: Biosensors for Virus Detection. Biosensors.

[17] Wang Y., Hu Y., He Q., Yan J., Xiong H., Wen N., Cai S., Peng D., Liu Y., Liu Z. (2020). Metal-organic frameworks for virus detection. Biosens. Bioelectron..

[18] Zaidi S.A., Cheong W.J. (2009). Preparation of an open-tubular capillary column with a monolithic layer of S-ketoprofen imprinted and 4-styrenesulfonic acid incorporated polymer and its enhanced chiral separation performance in capillary electrochroma-tography. J. Chromatogr. A.

[19] Jang R., Kim K.H., Zaidi S.A., Cheong W.J., Moon M.H. (2011). Analysis of phospholipids using an open-tubular capillary col-umn with a monolithic layer of molecularly imprinted polymer in electrochromatography-electrospray ionization-tandem mass spectrometry. Electrophoresis.

[20] Zaidi S.A. (2013). Dual-templates molecularly imprinted monolithic columns for the evaluation of serotonin and histamine in CEC. Electrophoresis.

[21] Zaidi S.A. (2014). Molecular imprinted polymers as drug delivery vehicles. Drug Deliv..

[22] Zaidi S.A. (2017). Molecular imprinting polymers and their composites: A promising material for diverse applications. Biomater. Sci..

[23] Zaidi S.A., Shin J.H. (2014). Molecularly imprinted polymer electrochemical sensors based on synergistic effect of composites synthe-sized from graphene and other nanosystems. Int. J. Electrochem. Sci..

[24] Pan G., Shinde S., Yeung S.Y., Jakštaitė M., Li Q., Wingren A.Q., Sellergren B. (2017). An epitope-imprinted biointerface with dy-namic bioactivity for modulating cell-biomaterial interactions. Angew. Chem. Int. Ed..

[25] Selvolini G., Marrazza G. (2017). MIP-Based Sensors: Promising New Tools for Cancer Biomarker Determination. Sensors.

[26] Teles F.S.R.R. (2011). Biosensors and rapid diagnostic tests on the frontier between analytical and clinical chemistry for biomolecular diagnosis of dengue disease: A review. Anal. Chim. Acta.

[27] Uzun L., Turner A.P. (2016). Molecularly-imprinted polymer sensors: Realising their potential. Biosens. Bioelectron..

[28] Cieplak M., Kutner W. (2016). Artificial Biosensors: How Can Molecular Imprinting Mimic Biorecognition?. Trends Biotechnol..

[29] Sharma P.S., Iskierko Z., Pietrzyk-Le A., D’Souza F., Kutner W. (2015). Bioinspired intelligent molecularly imprinted polymers for chemosensing: A mini review. Electrochem. Commun..

[30] Eersels K., Lieberzeit P., Wagner P. (2016). A Review on Synthetic Receptors for Bioparticle Detection Created by Surface-Imprinting Techniques—From Principles to Applications. ACS Sensors.

[31] Crapnell R.D., Hudson A., Foster C.W., Eersels K., Van Grinsven B., Cleij T.J., Banks C.E., Peeters M. (2019). Recent Advances in Electrosynthesized Molecularly Imprinted Polymer Sensing Platforms for Bioanalyte Detection. Sensors.

[32] Lowdon J.W., Diliën H., Singla P., Peeters M., Cleij T.J., van Grinsven B., Eersels K. (2020). MIPs for commercial application in low-cost sensors and assays—An overview of the current status quo. Sens. Actuators B Chem..

[33] Peeters M. (2015). Molecularly imprinted polymers (Mips) for bioanalytical sensors: Strategies for incorporation of Mips into sensing platforms. Austin J. Biosens. Bioelectron..

[34] Pan J., Chen W., Ma Y., Pan G. (2018). Molecularly imprinted polymers as receptor mimics for selective cell recognition. Chem. Soc. Rev..

[35] Altintas Z., Gittens M., Pocock J., Tothill I.E. (2015). Biosensors for waterborne viruses: Detection and removal. Biochimica.

[36] Afzal A., Mujahid A., Schirhagl R., Bajwa S.Z., Latif U., Feroz S. (2017). Gravimetric Viral Diagnostics: QCM Based Biosensors for Early Detection of Viruses. Chemosensors.

[37] Malik A.A., Nantasenamat C., Piacham T. (2017). Molecularly imprinted polymer for human viral pathogen detection. Mater. Sci. Eng. C.

[38] Sankarakumar N., Tong Y.W. (2013). Preventing viral infections with polymeric virus catchers: A novel nanotechnological approach to anti-viral therapy. J. Mater. Chem. B.

[39] Li N., Liu Y.-J., Liu F., Luo M.-F., Wan Y.-C., Huang Z., Liao Q., Mei F.-S., Wang Z.-C., Jin A.-Y. (2017). Bio-inspired virus imprinted polymer for prevention of viral infections. Acta Biomater..

[40] Altintas Z., Gittens M., Guerreiro A., Thompson K.-A., Walker J.A., Piletsky S.A., Tothill I.E. (2015). Detection of Waterborne Viruses Using High Affinity Molecularly Imprinted Polymers. Anal. Chem..

[41] Altintas Z., Pocock J., Thompson K.-A., Tothill I.E. (2015). Comparative investigations for adenovirus recognition and quantifica-tion: Plastic or natural antibodies?. Biosens. Bioelectron..

[42] Gast M., Kuehner S., Sobek H., Walther P., Mizaikoff B. (2018). Enhanced Selectivity by Passivation: Molecular Imprints for Viruses with Exceptional Binding Properties. Anal. Chem..

[43] Gast M., Sobek H., Mizaikoff B. (2019). Selective virus capture via hexon imprinting. Mater. Sci. Eng. C.

[44] Su C.-C., Wu T.-Z., Chen L.-K., Yang H.-H., Tai D.-F. (2003). Development of immunochips for the detection of dengue viral antigens. Anal. Chim. Acta.

[45] Babacan S., Pivarnik P., Letcher S., Rand A. (2000). Evaluation of antibody immobilization methods for piezoelectric biosensor application. Biosens. Bioelectron..

[46] Wu T.Z., Su C.C., Chen L.K., Yang H.H., Tai D.F., Peng K.C. (2005). Piezoelectric immunochip for the detection of dengue fever in viremia phase. Biosens. Bioelectron..

[47] Tai D.-F., Lin C.-Y., Wu T.-Z., Chen L.-K. (2005). Recognition of Dengue Virus Protein Using Epitope-Mediated Molecularly Imprinted Film. Anal. Chem..

[48] Tai D.-F., Lin C.-Y., Wu T.-Z., Huang J.-H., Shu P.-Y. (2006). Artificial Receptors in Serologic Tests for the Early Diagnosis of Dengue Virus Infection. Clin. Chem..

[49] Lieberzeit P., Chunta S., Navakul K., Sangma C., Jungmann C. (2016). Molecularly Imprinted Polymers for Diagnostics: Sensing High Density Lipoprotein and Dengue Virus. Procedia Eng..

[50] Laconi A., Fortin A., Bedendo G., Shibata A., Sakoda Y., Awuni J.A., Go-Maro E., Arafa A., Ali A.S.M., Terregino C. (2020). Detection of avian influenza virus: A comparative study of the in silico and in vitro performances of current RT-qPCR assays. Sci. Rep..

[51] Grabowska I., Malecka K., Jarocka U., Radecki J., Radecka H. (2014). Electrochemical biosensors for detection of avian influenza virus—current status and future trends. Acta Biochim. Pol..

[52] Liu X., Cheng Z., Fan H., Ai S. (2011). Electrochemical detection of avian influenza virus H5N1 gene sequence using a DNA ap-tamer immobilized onto a hybrid nanomaterial-modified electrode. Electrochim. Acta..

[53] Wangchareansak T., Thitithanyanont A., Chuakheaw D., Gleeson M.P., Lieberzeit P.A., Sangma C. (2013). Influenza A virus mo-lecularly imprinted polymers and their application in virus sub-type classification. J. Mater. Chem. B.

[54] Wangchareansak T., Thitithanyanont A., Chuakheaw D., Gleeson M.P., Lieberzeit P.A., Sangma C. (2014). A novel approach to identify molecular binding to the influenza virus H5N1: Screening using molecularly imprinted polymers (MIPs). Med. Chem. Commun..

[55] Karthik A., Margulis K., Ren K., Zare R.N., Leung L.W. (2015). Rapid and selective detection of viruses using virus-imprinted polymer films. Nanoscale.

[56] Enriquez C.E., Abbaszadegan M., Pepper I.L., Richardson K.J., Gerba C.P. (1993). Poliovirus detection in water by cell culture and nucleic acid hybridization. Water Res..

[57] Wang Y., Zhang Z., Jain V., Yi J., Mueller S., Sokolov J., Liu Z., Levon K., Rigas B., Rafailovich M.H. (2010). Potentiometric sensors based on surface molecular imprinting: Detection of cancer biomarkers and viruses. Sens. Sens. Actuators B Chem..

[58] Ozer T., Geiss B.J., Henry C.S. (2020). Review—Chemical and Biological Sensors for Viral Detection. J. Electrochem. Soc..

[59] Yang B., Gong H., Chen C., Chen X., Cai C. (2017). A virus resonance light scattering sensor based on mussel-inspired molecularly imprinted polymers for high sensitive and high selective detection of Hepatitis A Virus. Biosens. Bioelectron..

[60] Luo L., Feng W., Hu W., Chen C., Gong H., Cai C. (2019). Molecularly imprinted polymer based hybrid structure SiO2@MPS-CdTe/CdS: A novel fluorescence probe for Hepatitis A virus. Methods Appl. Fluoresc..

[61] Luo L., Zhang F., Chen C., Cai C. (2020). Molecular imprinting resonance light scattering nanoprobes based on pH-responsive metal-organic framework for determination of hepatitis A virus. Microchim. Acta.

[62] Luo L., Zhang F., Chen C., Cai C. (2019). Visual Simultaneous Detection of Hepatitis A and B Viruses Based on a Multifunctional Molecularly Imprinted Fluorescence Sensor. Anal. Chem..

[63] He K., Chen C., Liang C., Liu C., Yang B., Chen X., Cai C. (2016). Highly selective recognition and fluorescent detection of JEV via virus-imprinted magnetic silicon microspheres. Sens. Sens. Actuators B Chem..

[64] Liang C., Wang H., He K., Chen C., Chen X., Gong H., Cai C. (2016). A virus-MIPs fluorescent sensor based on FRET for highly sensitive detection of JEV. Talanta.

[65] Yang J., Feng W., Liang K., Chen C., Cai C. (2020). A novel fluorescence molecularly imprinted sensor for Japanese encephalitis virus detection based on metal organic frameworks and passivation-enhanced selectivity. Talanta.

[66] Luo L., Yang J., Liang K., Chen C., Chen X., Cai C. (2019). Fast and sensitive detection of Japanese encephalitis virus based on a magnetic molecular imprinted polymer–resonance light scattering sensor. Talanta.

[67] Feng W., Liang C., Gong H., Cai C. (2018). Sensitive detection of Japanese encephalitis virus by surface molecularly imprinted tech-nique based on fluorescent method. N. J. Chem..

[68] Bai W., Spivak D.A. (2014). A double-imprinted diffraction-grating sensor based on a virus-responsive super-aptamer hydrogel de-rived from an impure extract. Angew. Chem. Int. Ed..

[69] Jenik M., Schirhagl R., Schirk C., Hayden O., Lieberzeit P., Blaas D., Paul G., Dickert F.L. (2009). Sensing Picornaviruses Using Molecular Imprinting Techniques on a Quartz Crystal Microbalance. Anal. Chem..

[70] Hussein H.A., Hassan R., El Nashar R.M., Khalil S.A., Salem S.A., El-Sherbiny I.M. (2019). Designing and fabrication of new VIP biosensor for the rapid and selective detection of foot-and-mouth disease virus (FMDV). Biosens. Bioelectron..

[71] Cumbo A., Lorber B., Corvini P.F.-X., Meier W., Shahgaldian P. (2013). A synthetic nanomaterial for virus recognition produced by surface imprinting. Nat. Commun..

[72] Cumbo A., Corvini P.F.-X., Shahgaldian P. (2013). A Novel Synthetic Virus Recognition Nanomaterial for Diagnostic and Environ-mental Applications. Chimia.

[73] Jablonski M., Poghossian A., Severins R., Keusgen M., Wege C., Schöning M.J. (2021). Capacitive Field-Effect Biosensor Studying Adsorption of *Tobacco Mosaic Virus* Particles. Micromachines.

[74] Hayden O., Bindeus R., Haderspock C., Mann K.-J., Wirl B., Dickert F.L. (2003). Mass-sensitive detection of cells, viruses and en-zymes with artificial receptors. Sens. Actuators B.

[75] Dickert F.L., Hayden O., Bindeus R., Mann K.-J., Blaas D., Waigmann E. (2004). Bioimprinted QCM sensors for virus detection—Screening of plant sap. Anal. Bioanal. Chem..

[76] Hayden O., Lieberzeit P.A., Blaas D., Dickert F.L. (2006). Artificial antibodies for boanalyte detection-sensing viruses and proteins. Adv. Funct. Mater..

[77] Bolisay L.D., Culver J.N., Kofinas P. (2006). Molecularly imprinted polymers for tobacco mosaic virus recognition. Biomaterials.

[78] Bolisay L.D., Culver J.N., Kofinas P. (2007). Optimization of virus imprinting methods to improve selectivity and reduce nonspecif-ic binding. Biomacromolecules.

[79] Bolisay L.D., Kofinas P. (2010). Imprinted Polymer Hydrogels for the Separation of Viruses. Macromol. Symp..

[80] Birnbaumer G.M., Lieberzeit P.A., Richter L., Schirhagl R., Milnera M., Dickert F.L., Bailey A., Ertl P. (2009). Detection of viruses with molecularly imprinted polymers integrated on a microfluidic biochip using contact-less dielectric microsensors. Lab a Chip.

[81] Ikawa T., Kato Y., Yamada T., Shiozawa M., Narita M., Mouri M., Hoshino F., Watanabe O., Tawata M., Shimoyama H. (2010). Virus-Templated Photoimprint on the Surface of an Azobenzene-Containing Polymer. Langmuir.

[82] Wankar S., Turner N.W., Krupadam R.J. (2016). Polythiophene nanofilms for sensitive fluorescence detection of viruses in drinking water. Biosens. Bioelectron..

[83] DiCaprio E. (2017). Recent advances in human norovirus detection and cultivation methods. Curr. Opin. Food Sci..

[84] Sykora S., Cumbo A., Belliot G., Pothier P., Arnal C., Dudal Y., Corvini P.F.-X., Shahgaldian P. (2015). Virus-like particles as virus substitutes to design artificial virus-recognition nanomaterials. Chem. Commun..

[85] Blome S., Staubach C., Henke J., Carlson J., Beer M. (2017). Classical Swine Fever—An Updated Review. Viruses.

[86] Klangprapan S., Choke-Arpornchai B., Lieberzeit P.A., Choowongkomon K. (2020). Sensing the classical swine fever virus with molecularly imprinted polymer on quartz crystal microbalance. Heliyon.

[87] Tancharoen C., Sukjee W., Thepparit C., Jaimipuk T., Auewarakul P., Thitithanyanont A., Sangma C. (2019). Electrochemical biosensor based on surface imprinting for Zika virus detection in derum. ACS Sens..

[88] Ding X., Heiden P.A. (2013). Recent Developments in Molecularly Imprinted Nanoparticles by Surface Imprinting Techniques. Macromol. Mater. Eng..

[89] Pokhrel P., Hu C., Mao H. (2020). Detecting the Coronavirus (COVID-19). ACS Sens..

[90] Ekrami E., Pouresmaieli M., Barati F., Asghari S., Ziarani F.R., Shariati P., Mamoudifard M. (2020). Potential Diagnostic Systems for Coronavirus Detection: A Critical Review. Biol. Proced. Online.

[91] Taha B.A., Al Mashhadany Y., Mokhtar M.H.H., Bin Zan M.S.D., Arsad N. (2020). An Analysis Review of Detection Coronavirus Disease 2019 (COVID-19) Based on Biosensor Application. Sensors.

